# A preliminary investigation of the relationship between water quality and *Anopheles gambiae* larval habitats in western Cameroon

**DOI:** 10.1186/1475-2875-12-225

**Published:** 2013-07-02

**Authors:** Michelle R Sanford, Steven Ramsay, Anthony J Cornel, Clare D Marsden, Laura C Norris, Salomon Patchoke, Etienne Fondjo, Gregory C Lanzaro, Yoosook Lee

**Affiliations:** 1 Harris County Institute of Forensic Sciences, Houston, TX, USA; 2 Vector Genetics Laboratory, School of Veterinary Medicine, University of California, Davis, CA, USA; 3 Department of Pathology, Microbiology and Immunology, University of California, Davis, CA, USA; 4 Department of Entomology and Nematology, University of California, Davis, CA, USA; 5 Ministry of Public Health, Yaoundé, Cameroon

**Keywords:** Conductivity, Total Dissolved Solids, Molecular Form, *Anopheles Gambiae* s.s

## Abstract

**Background:**

Water quality and anopheline habitat have received increasing attention due to the possibility that challenges during larval life may translate into adult susceptibility to malaria parasite infection and/or insecticide resistance.

**Methods:**

A preliminary study of *Anopheles gambiae* s.s. larval habitats in the north-west and south-west regions of Cameroon was conducted in order to detect associations between *An. gambiae* s.s. molecular form and *2La* inversion distributions with basic water quality parameters. Water quality was measured by temperature, pH, conductivity, total dissolved solids (TDS) at seven sites in Cameroon and one site in Selinkenyi, Mali.

**Results:**

Principal components and correlation analyses indicated a complex relationship between *2La* polymorphism, temperature, conductivity and TDS. Cooler water sites at more inland locations yielded more S form larvae with higher *2La* inversion polymorphism while warmer water sites yielded more M form larvae with rare observations of the *2La* inversion.

**Discussion:**

More detailed studies that take into account the population genetics but also multiple life stages, environmental data relative to these life stages and interactions with both humans and the malaria parasite may help us to understand more about how and why this successful mosquito is able to adapt and diverge, and how it can be successfully managed.

## Background

As the primary vector of *Plasmodium falciparum* in West Africa, *Anopheles gambiae* s.s. is among the best-studied malaria vector species in Africa. A large literature exists from many of countries in West Africa, where population genetics [1-5] and genomics [6-11] have revealed population structure and differentiation among populations of *An. gambiae* s.s., including the molecular forms of M and S [2], and the five chromosomal forms (Mopti, Savanna, Bamako, Bissau and Forest), which are characterized by different arrangements of a set of six paracentric inversions [12-16].

The factors underlying divergence within *An. gambiae* have been the subject of much interest. Despite low frequencies of molecular form hybrids and among chromosomal forms [17,18], studies suggest an absence of intrinsic post-mating reproductive isolation mechanisms [19,20], indicating that pre-mating factors play a major role in the evolution and maintenance of divergence. Whilst specific mechanisms underlying divergence among chromosomal forms have not been identified, the forms have been associated with particular habitats, and thus ecologically based divergence is thought to be important [21,22]. In the molecular forms, considerable evidence supports the importance of a number of pre-mating factors including mating swarm segregation [23], cuticular hydrocarbon differentiation [24], wing beat frequency harmonization [25,26] and larval habitat segregation [27-29]. Until recently, these have been largely assumed to be universal throughout the large distribution of the species [30]. However, recent work in Guinea-Bissau, has revealed both genetic [31,32] and phenotypic [26] departures from previous assumptions. Larval habitat segregation between the molecular forms shares its foundation with the ecologically based chromosomal speciation hypothesis which suggests that differing ecological characteristics are driving divergence and adaptation among populations of *An*. *gambiae*[21,33,34]. A recent study in Burkina Faso supports this hypothesis by showing differential habitat utilization between the M and S forms in agricultural rice fields and natural habitats [29,35]. In Burkina Faso, M forms are more often encountered in rice fields whereas S forms are more often found in naturally occurring puddles [29,35].

Breaking with previous assumptions, recent work in Cameroon has shown a more complicated pattern of habitat segregation. Samples collected from developed areas of the country identified more S form larvae in cultivated areas (not necessarily associated with rice) whereas M form larvae were more often encountered in natural pools (both polluted and unpolluted) in the urbanized areas of Yaoundé and Douala [36]. In the southern agricultural area of Niete the M form predominates [37]. One potential explanation for the differences is that the relationship between molecular forms and chromosomal forms is complicated by further population structure in Cameroon. Specifically, the Forest-M form, which is characterized by the absence of chromosome inversions, has been identified as a separate subpopulation within the M form [3,38]. Moreover, the Forest-M form has been shown to be associated with the humid evergreen forest habitat in contrast to the Mopti-M form which occurs most commonly in dry environments [9,27] and carries *2R b, c* and *u* inversions [34].

Consistent with research in other taxa [39-43], specific chromosomal inversions in *An. gambiae* have shown to be strongly correlated with ecological parameters. In Cameroon, the large inversion on the left arm of the second chromosome, *2La*, which is found in the all chromosomal forms except Forest, has been associated with desiccation resistance in adults [44,45] and is often associated with populations in arid areas of West Africa. Within the S form the *2La* inversion follows a latitudinal cline with the inversion predominating in the drier north of Cameroon and rarely occurring in the tropical south of Cameroon [46].

The goal of this study was to assess patterns of molecular form and *2La* inversion polymorphism in north-western Cameroon with basic water quality parameters. The location of the sites in this study covers both areas where the *2La* inversion is polymorphic and molecular forms are heterogeneous to explore the potential for patterns related to water quality, *2La* inversion and molecular form.

## Methods

### Collection

Anopheline larvae were collected from characteristic aquatic habitats near human habitations at seven sites in Cameroon with an additional site from Mali included for comparative purposes (Table 1). A minimum of ten larvae were collected at each site in conjunction with the water quality parameters of temperature, conductivity, total dissolved solids (TDS), and pH as measured with a Hanna Instruments HI 9812–5 (Ann Arbor, MI, USA). Calibration was conducted with Hanna Instruments calibration solutions (pH: HI 7004, HI 7007, HI 7010; Conductivity: HI 70031; TDS: HI 70032) before each measurement was made. The measurements were taken at the same time of day, approximately 11:00 AM. Table 1 lists the site locations and their respective water quality measurements. Diabate and his colleagues suggested the effect of mosquito larval predators in divergence between M and S forms [47]. Thus we logged our observation regarding larval predators such as beetles, dragonfly nymphs or aquatic hemipterans for each sampling location. Larvae were reared to adults in the field sites when possible to facilitate sex determination as well as to differentiate predatory mosquito larvae from *An. gambiae*. All specimens were preserved in 70% ethanol.

**Table 1:** **Collection site locations, date of measurement and water quality parameters of temperature (Temp.), conductivity (Cond.), total dissolved solids (TDS), pH and *An. gambiae *s.l. sample size (N) measured at seven sites in Cameroon and a single site in Mali**

**Site**	**Lat.**	**Long.**	**Collection date**	**Temp. (°C)**	**Cond. (μS/cm)**	**TDS (ppm)**	**pH**	**N**
Wasi-Ber	6.20	10.67	28-Aug-11	21	150	60	7.1	11
Bamessing	5.63	10.23	30-Aug-11	24	610	350	8.6	14
New Pongo	4.12	9.41	5-Sept-11	28.5	190	95	6.9	15
Sonné	4.10	9.31	2-Sept-11	28.5	520	270	7.3	81
Small Ikangé	4.09	9.37	6-Sept-11	31	310	170	6.7	13
Near river outlet, Tiko	4.07	9.37	3-Sept-11	35	300	150	8.7	15
Limbé	4.01	9.19	4-Sept-11	29	30	20	6.2	22
Selinkenyi, Mali	11.7	−8.28	29-Sept-11	38	110	60	6.8	10

### Processing

Each individual mosquito was processed for DNA extraction with the Qiagen (Valencia, CA, USA) Blood and Tissue kit adapted for the high-throughput Biosprint 96® system with tissue disruption using a Qiagen Tissuelyzer® following the manufacturer’s instructions. Species determinations were made via PCR to distinguish between members of the *An*. *gambiae* complex following Scott *et al.*[48]. Molecular form determinations of *An*. *gambiae* s.s. were made using the method of Favia *et al.*[49] followed by the method of Santolamazza *et al.*[50] to clarify samples that gave ambiguous results with the first PCR.

Lee *et al.*[3] suggested that molecular form combined with *2La* inversion data provides good proxy for differentiating the Forest-M form from Mopti-M form. Thus, the *2La* inversion status of each mosquito was determined by PCR using the method of White *et al.*[51]. Insertion-deletion polymorphisms were observed in the PCR fragment patterns of hetero- and homokaryotypic individuals as described by Obbard *et al.*[52]. Finally the sex of any larval mosquitoes remaining in the samples was determined with the PCR method of Ng’habi *et al.*[53]. The resulting PCR products were analysed with agarose gels specific to their respective methods or with the use of the Qiagen Qiaexcel® DNA screening cartridge capillary system and the Biocalculator® software using the AM420 method.

### Analysis

The site GPS coordinates, water quality data, PCR results of *2La* polymorphism and the sex of each mosquito were used to construct a principal components analysis using the Multibase2013 (NumericalDynamics.com) Excel 2010 add-in (Microsoft Inc, Redmond, WA, USA). In addition, a set of Pearson’s product moment correlations were conducted among the variables of interest, using SPSS 16.0 [54]. The significance level of all statistical tests was observed at α = 0.05.

## Results

**Figure 1:**
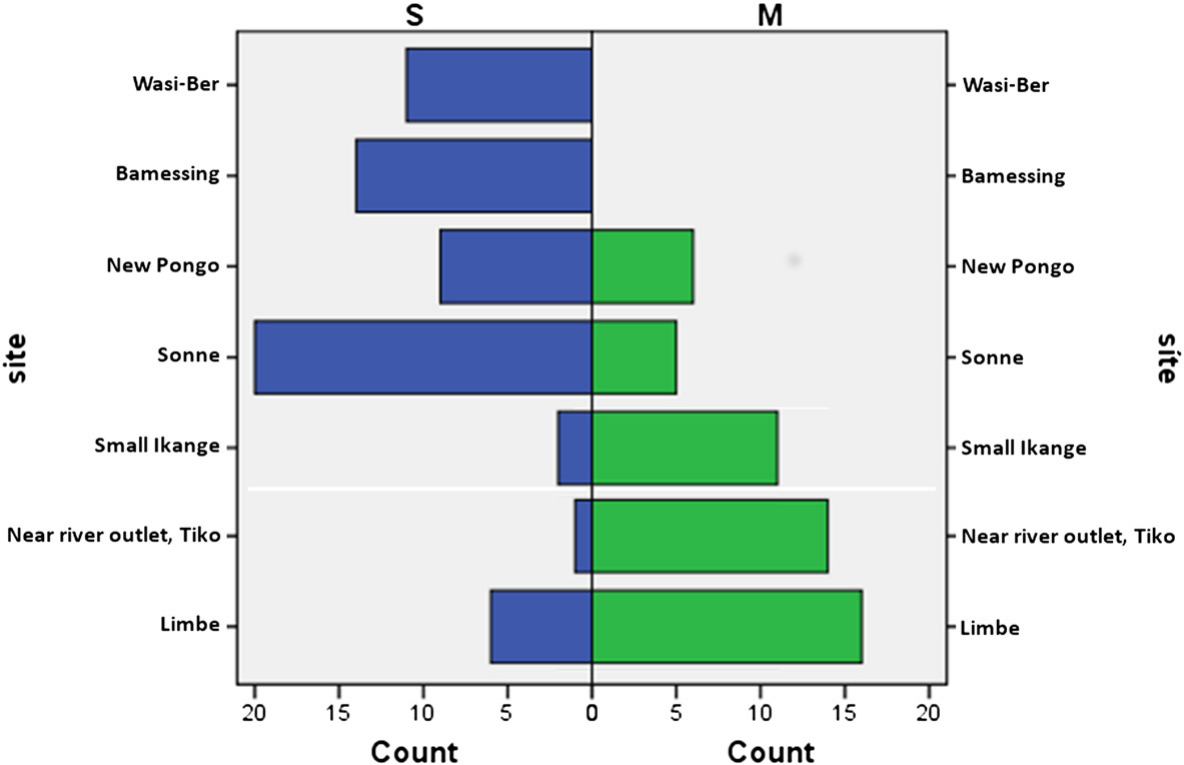
**Molecular form distribution of larvae collected during the survey or larval habitats in north-western Cameroon in August/September 2011.** Collection site is listed by latitude in descending order.

**Figure 2:**
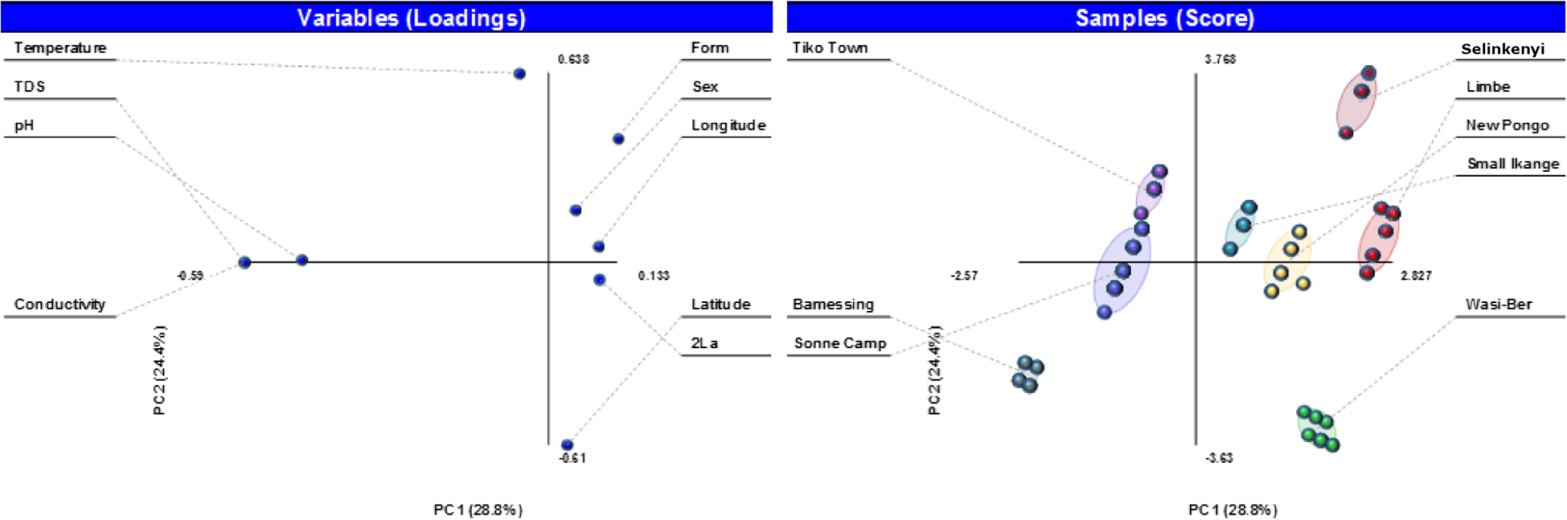
**Principal component analysis (PCA), with variable loadings, illustrating the relationships between the variables measured in the current study investigating the relationship between water quality, *2La *inversion polymorphism, sex and molecular form in north-western Cameroon.**

All larval samples were determined to be *An*. *gambiae* s.s. Figure 1 illustrates the distribution of M and S molecular forms by site. No M/S hybrids were detected in the larval collections. The S form comprised the majority of collections made in cooler temperature water, while the M form was most commonly collected in warmer temperature habitats located near the coast (Figure 2). Molecular form (coded as 0 for S and 1 for M) was significantly correlated with site (latitude and longitude) and temperature (Table 2), which were also correlated with each other.

**Table 2:** Pearson’s product moment correlations among the variables measured in the larval habitat survey conducted in north-western Cameroon

	**Longitude**	**Latitude**	**Temp.**	**pH**	**Cond.**	**TDS**	**Form**	***2La***	**Sex**
Long.	**1.000**	<0.001	<0.001	<0.001	0.036	0.032	<0.001	<0.001	0.005
Lat.	0.993^**^	**1.000**	<0.001	<0.001	0.036	0.039	<0.001	<0.001	0.004
Temp.	−0.817^**^	−0.779^**^	**1.000**	0.266	0.124	0.107	<0.001	<0.001	0.074
pH	0.331^**^	0.361^**^	**0.104**	**1.000**	<0.001	<0.001	0.162	0.217	0.018
Cond.	0.196^*^	0.195^*^	**−0.144**	0.655^**^	**1.000**	<0.001	<0.001	0.980	0.180
TDS	0.200^*^	0.193^*^	**−0.151**	0.646^**^	0.995^**^	**1.000**	<0.001	0.964	0.217
Form	−0.484^**^	−0.467^**^	0.612^**^	**−0.131**	−0.359^**^	−0.349^**^	**1.000**	0.001	0.128
*2La*	0.654^**^	0.655^**^	−0.555^**^	**0.117**	**−0.002**	**−0.004**	−0.320^**^	**1.000**	0.094
Sex	−0.257^**^	−0.263^**^	**0.167**	−0.220^*^	**−0.126**	**−0.116**	**0.143**	**−0.158**	**1.000**

Upper triangle matric indicates P-values and lower triangle matric indicate Pearson Correlation. Temp. stands for Temperature and Cond. for conductivity.

**. Correlation is significant at the 0.01 level (2-tailed).

*. Correlation is significant at the 0.05 level (2-tailed).

**Figure 3:**
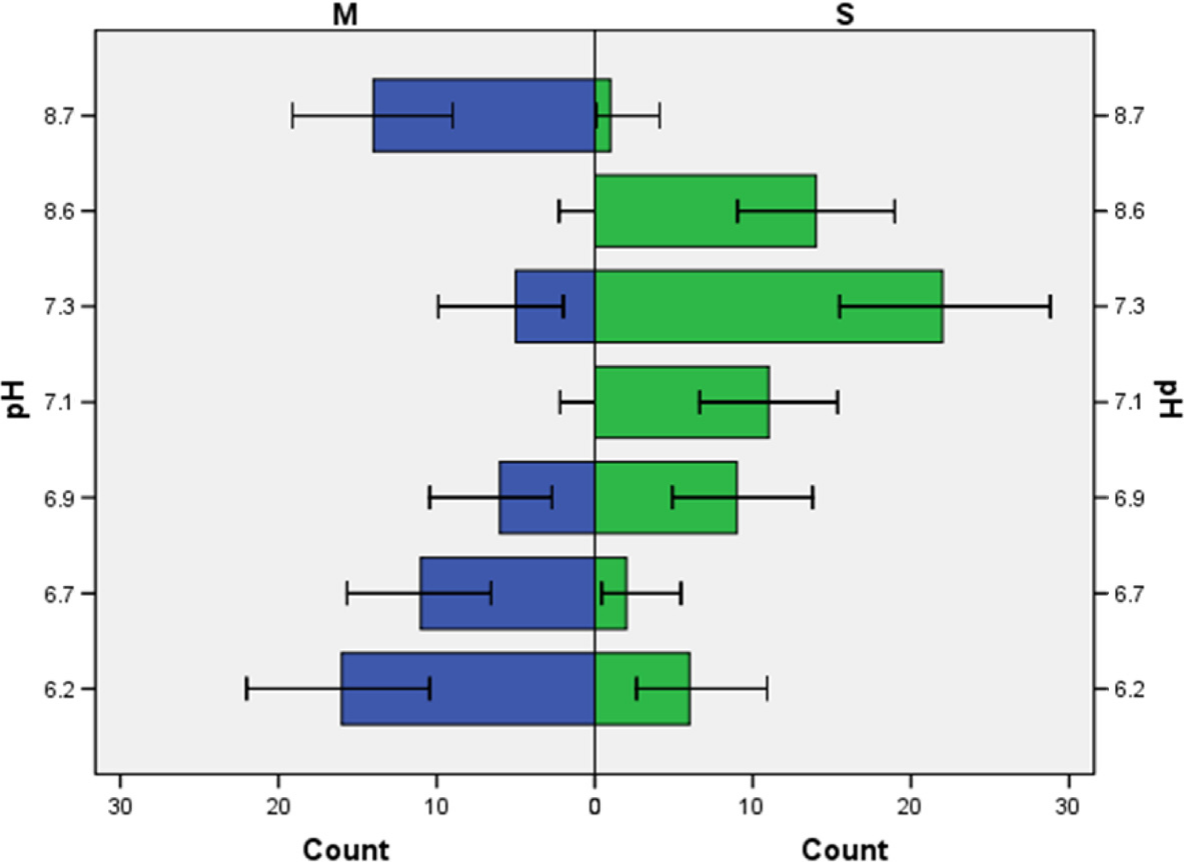
**Distribution of individuals of each molecular form by water pH in north-western Cameroon.** Bars indicate 95% confidence intervals.

Between villages, pH levels varied from 6.2 to 8.7 (Table 1), with the M form more common at the extremes of this range, while the S form was more common around neutral pH (Figure 3). Previous studies have shown subdivision within the M form populations in Cameroon [3,38], which raises the question of whether the M form collected from lower pH pools differed from the M form collected from pools of higher pH. However, no correlations between 2La inversion polymorphism and pH were detected. Thus population subdivision does not appear to be a cause factor in pH preference of M form. Rather, these data suggest it may be due to the M form having a better buffer resistance to pH changes.

The S molecular form was also significantly correlated with the presence of the *2La* inversion while the M form, collected in warmer water did not display *2La* polymorphism. The interconnected nature of this relationship between these factors is illustrated in Figure 2. While none of the samples was karyotyped for other inversions, the lack of *2La* polymorphism in the mosquitoes near the coast strongly suggests the presence of the Forest-M chromosomal form. The relationship between the M form and both conductivity and TDS was also significant with a negative correlation observed between these water quality parameters and the presence of the M form (Table 2). All of the larvae collected from Mali were homozygous for the *2La* inversion.

As evident in Table 1, there were relatively large gap in sampling locations between Bamessing and New Pongo. Our collection attempt was hampered by two factors: (1) the poor condition of the roads that made travel exceedingly difficult in the rainy season and (2) the large amount of rain flushed larval habitats. We sampled several sites between Bamessing and New Pongo and failed to find larvae, presumably because the rains were flushing the habitats as fast as they were being colonized during our collection trip in late August and early September.

There were no known predatory insects observed in the pools the mosquitoes were collected from at the time of collections. No beetles, dragonfly nymphs or aquatic hemipterans were observed. However, it is possible that predatory mosquito larvae may have been present as they would have been impossible to differentiate by sight. However, no predatory mosquito larvae were collected in the reared and preserved samples. The correlation between the larval sex ratio and temperature was not statistically significant (χ^2^ = 11.433, d.f. = 6, P = 0.076).

## Discussion

In this small study of larval *An*. *gambiae* habitats in north-western Cameroon, several water quality parameters were found to be significantly correlated with molecular forms. Male and female *An*. *gambiae* s. s. of the M and S molecular forms with a range of *2La* inversion polymorphism, were collected from typical larval habitats near human dwellings. The S molecular form was collected in cooler water with higher conductivity and TDS and was characterized by variation in the *2La* inversion. The M molecular form was collected in warmer water with lower conductivity and TDS and was characterized by the rarity of the *2La* inversion. These findings are consistent with niche differentiation, and suggest the important role of water temperature in habitat preference.

The strong correlation observed between conductivity and TDS in the current study is similar to the result obtained by Edillo *et al.*[28] for young larvae collected in Banambani, Mali. They found that conductivity and TDS of larval habitat had significant effects on the relative proportions of M and S form larvae. Both the M and S forms in Mali are polymorphic but nearly fixed for the *2La* inversion [55]. In the study presented here, those larvae polymorphic for the *2La* inversion were also associated with higher conductivity and TDS. As Edillo *et al.*[28] point out, conductivity and TDS are related to each other and are in fact proportionally related and affected by temperature [56]. Thus, the significant correlations observed in the present study between temperature, conductivity and TDS in relation to molecular form might reflect adaptation to a specific combination of inter-related larval habitat characteristics. More widespread sampling among sites to establish site variation would help to establish characteristics of larval habitats and *An*. *gambiae* molecular forms in this part of Cameroon.

The relationship between environmental temperature and M and S distribution has been correlated with adult *An*. *gambiae* s.s. in Cameroon. Simard *et al.*[57] found significant correlations between air temperature and M and S distribution among other environmental variables in Cameroon. In the current study, the temperature of the larval habitat was significantly correlated with M and S form larvae. Data collected in Burkina Faso have also suggested a strong correlation between M and S form larval habitat segregation and larval habitat characteristics [29]. Strong correlations were found between rice agriculture and M form mosquitoes. The relationship between water temperature of the larval habitat and air temperature often collected with climatic variables is not necessarily linear. In Kenya, Paaijamans *et al.*[58] found that water temperature of artificial mosquito larval habitats was consistently higher than air temperature. Therefore correlating temperatures with form distribution is complicated by the effect of temperature on the different life stages and its relationship to other environmental factors that also have differential effects on life stage (e.g., humidity).

The effect of temperature as it relates to sex ratio was investigated in laboratory rearing of *An. gambiae* and *Anopheles arabiensis* in mixed and monocultures [59]. As temperatures increased from 25 to 35°C the ratio of females to males decreased. Observed in the current study, however, did not show significant correlation with sex ratio and temperature. The current study differs in that it represents a single time point in the lives of field-collected mosquitoes from fluctuating natural temperature regimes rather than constant temperature rearing as in the laboratory rearing study. Fluctuating rearing temperatures affect sex ratios at higher temperatures in *Aedes aegypti*[60]. However, more detailed field measurements of temperature with respect to life stage will allow for better understanding of how and at what age temperature affects sex and survival.

Previous studies in Cameroon have noted strong north–south clinal variation in the *2La* inversion that follows an environmental moisture gradient that runs from the moist south to arid north [46]. In addition, polymorphism in various chromosomal inversions was correlated with the S form along a similar north–south cline [57]. Simard *et al.*[57] also found less inversion polymorphism in the M form. Despite the small scale of the current study, a similar result was observed with more polymorphism in the *2La* inversion noted in the S form and less in the M form. The current study also supports the proposed habitat suitability maps created by Simard *et al.*[57] for Cameroon, where more M forms were collected near the Atlantic coast than in the more inland and northern areas.

Although not karyotyped for inversions on 2R, the lack of *2La* polymorphism and an abundance of M form near the Atlantic coast of Cameroon suggest the presence of the Forest-M form. Lee *et al.*[3] also found many Forest-M form samples in the same areas sampled in the current study and provided evidence suggesting the *2La* inversion and molecular form provides a good proxy for Forest-M form in southern Cameroon in the absence of karyotype data. The population subdivision within the M form combined with the limited scale of the current study [3,38] complicates the interpretation of the findings presented here. Specifically, since the majority of M form samples also did not possess the *2La* inversion one cannot rule out the possibility that the water quality factors correlated with the M form were related specifically to the Forest-M form, and are not representative of M form in general in Cameroon. The lower number of samples collected from sites where the *2La* inversion is more polymorphic were not sufficient to overcome this confounding factor. A larger scale sampling effort would help to address the confounding nature of the Forest-M form.

Water quality and anopheline habitat have also received increasing attention due to the possibility that stress during larval life may translate into adult susceptibility to malaria parasite infection [61] and/or insecticide resistance [36]. Okech *et al.*[61] showed that the presence of bacteria in the larval habitat resulted in more pupation and slightly larger individual *An*. *gambiae* than those reared in sterile substrate, strongly suggesting a role for microbes in larval habitats [61]. Autoclaving and killing off bacteria did not appear to affect infection rate in adults exposed to *Plasmodium falciparum*. However, soil type had a small effect, suggesting that larval habitat affects malaria infection susceptibility. The influence of pollution and agricultural impact on larval habitat has been suggested to have a strong effect on insecticide resistance in the agricultural areas of southern Cameroon [36]. In a detailed study of M and S larval habitats near Yaoundé, Cameroon, Kamdem *et al.*[62] found that the M form is becoming increasingly capable of survival in polluted habitats.

Taking into consideration the complicated nature of the various mechanisms driving population divergence in *An*. *gambiae* in Cameroon, understanding the ecological source of population divergence and mosquito control continues to be a challenge. More detailed studies that take into account the population genetics, multiple life stages, environmental data, and interactions with both humans and the malaria parasite may help us to understand more about how and why this mosquito has successfully adapted and diverged, and how it can be successfully managed.

## Competing interests

The authors declare that they have no competing interests.

## Authors’ contributions

MRS, AJC, SP carried out field collection of mosquito specimens. GCL and EF provided logistical support for field collection and molecular genetic studies. SR conducted the molecular genetic studies. MRS, CDM, LCN, GCL and YL drafted the manuscript. MRS participated in the design of the study and performed the statistical analysis. All authors read and approved the final manuscript.
